# An Ensemble-Based Multiclass Classifier for Intrusion Detection Using Internet of Things

**DOI:** 10.1155/2022/1668676

**Published:** 2022-05-20

**Authors:** Deepti Rani, Nasib Singh Gill, Preeti Gulia, Jyotir Moy Chatterjee

**Affiliations:** ^1^Department of Computer Science & Applications, Maharshi Dayanand University, Rohtak, Haryana, India; ^2^Department of Information Technology, Lord Buddha Education Foundation, Kathmandu, Nepal

## Abstract

Internet of Things (IoT) is the fastest growing technology that has applications in various domains such as healthcare, transportation. It interconnects trillions of smart devices through the Internet. A secure network is the basic necessity of the Internet of Things. Due to the increasing rate of interconnected and remotely accessible smart devices, more and more cybersecurity issues are being witnessed among cyber-physical systems. A perfect intrusion detection system (IDS) can probably identify various cybersecurity issues and their sources. In this article, using various telemetry datasets of different Internet of Things scenarios, we exhibit that external users can access the IoT devices and infer the victim user's activity by sniffing the network traffic. Further, the article presents the performance of various bagging and boosting ensemble decision tree techniques of machine learning in the design of an efficient IDS. Most of the previous IDSs just focused on good accuracy and ignored the execution speed that must be improved to optimize the performance of an ID model. Most of the earlier pieces of research focused on binary classification. This study attempts to evaluate the performance of various ensemble machine learning multiclass classification algorithms by deploying on openly available “TON-IoT” datasets of IoT and Industrial IoT (IIoT) sensors.

## 1. Introduction

For the last few decades, Internet of Things (IoT) technology has been continuously integrated with various application domains, especially in design of automation-enabled homes, cities, and industries. A plethora of physical and virtual “things” communicate with each other using the Internet. IoT has become a usual chunk of people's lives and it is expanding rapidly due to its capability of providing superior services. IoT has improved people's everyday lives by automating very common home services such as controlling the temperature of refrigerators, turning on/off light bulbs, operating ACs, and locking/unlocking doors. IoT has reshaped even the modern technologies with the absolute connection of things in various domains, namely, home, industry, and business [1]. However, due to the frequent rising of IoT technology, it is exposed to many technical and security challenges [2]. The Internet of Things is described as a group of physical objects and applications that are embedded with several components including sensors, actuators, software, processors, and many other technologies and services which enable devices and systems to connect and communicate with other devices over the Internet using a communication network. The sensors and actuators embedded with devices collect valuable information from the related environment of the physical world and transmit it over the network. Data gathered from these automated devices flow in the form of signals which might carry suspected network traffic along with the normal network traffic. The traffic signals flowing over the network might be stored on different levels of IoT platform. Data storage might occur on network, cloud, fog, or device itself, and unauthorized users might smartly access the whole system through anomalous traffic signals [3, 4]. IoT devices could be easily compromised by malicious users by merging anomalous traffic with normal traffic. An attacker can easily access the user's login and trace his activities to figure out confidential information [5].

Every device operates in a specific pattern. If a user tries to operate any device differently, that results in a change in the normal behavior of that device, and the modified behavior is considered as malicious [6]. In such situations, the current behavior is matched with the historical behavior of the specific device to verify the mode of behavior (whether safe or unsafe). Intrusions could be identified by recognizing changes that happened in the behavior of events [7]. The actual authorized user could be alerted if such kind of changes occurs. In IoT-enabled smart home environment, the attackers can make physical effects on various devices like smart refrigerators, smart doorbells, fire alarms, smart heaters, domestic useable smart healthcare devices, etc. If these devices are controlled by malicious users, they can make changes in the behavior of these devices. By gaining unauthorized home access, the attackers can disrupt the power supply, close and open door locks without the user's permission, and give wrong instructions to a smart refrigerator or a heater, which may cause massive hazards [8]. Attacks on computers are generally limited to data loss, but attacks on IoT systems might result in the loss of data as well as loss of someone's life too.

The malicious access could be prevented by robust security schemes. In this context, there are several major methods that have been gaining the remarkable attention of researchers. Handling of any cybercrime-related problem should be started with the intrusion detection system (IDS). Intrusion detection (ID) is the most effective mechanism to detect the mode of a device's behavior [9]. Illustration of the user's behavioral pattern and secure cyber systems might lead to anomalous behavior detection [10]. Intrusion detection is a process of scanning the incidents which arise in different network systems and examining them to find clues related to incidents [11]. It is an effective technique that can reduce the growing cybersecurity issues through the proactive security system. So far, IDSs proposed by different researchers have achieved remarkable results to predict known and unknown intrusions in wired and wireless IoT networks. The network traffic might be examined to understand the properties of the attacks and the transmission medium [12]. The main contributions of this study are as follows:

- This study provides a diversified evaluation of network traffic routines in IoT enabled smart environment.
- This study also highlights the review of various decision tree ensemble techniques for the classification of network traffic data of IoT systems.
- This study adopts various classification metrics to predict malicious network traffic as well as normal traffic with the help of collected data patterns.
- The work in this study also focuses on the comparison of various ensemble learning techniques for multiclass classification and threat detection in IoT environment.

For practical implementation, the “TON_IoT” datasets have been accessed from an open-access location [13]. Here, a CSV file of “TON_IoT” has been downloaded that includes heterogeneous data sources collected from telemetry datasets of IoT and IIoT sensors. It has been classified using various multiclass classifiers to predict the labels by training the model with training data samples. The proposed model is trained using the collected datasets and the extracted features. In a smart IoT environment, a particular device generally operates in a unique pattern. In this article, Section 2 presents the motivation and related work to the proposed work.  Section 3 presents the methods and techniques used for the proposed work. Bagging and boosting ensemble approaches have been explored thoroughly for multiclass classification for the purpose of ID.  Section 4 comes with details of the considered datasets and the procedure followed for dataset selection used in the practical implementation.  Section 5 comprises the experimental results and metrics with a brief discussion, computation, and analysis.

## 2. Motivation and Related Work

IoT is an emerging technology that is growing day by day. IoT system's users have to face various unexpected situations. There are numerous challenges in the path of successful IoT infrastructure. Smart devices are the major pillars of IoT infrastructure. The things of daily needs, which are positioned in domestic, industrial, and other application areas, are now interconnected with the Internet and are enabled with very few security measures. Therefore, the security and privacy of the IoT environment are really unpredictable. The interconnected IoT systems generate an amount of digital data related to the objects, applications, and their behavior. The generated data needs to be collected, processed, analyzed, and distributed securely and efficiently.

In view of the increasing number of cybercrimes in connected devices, it is required to formulate new generation approaches to identify the classes of intrusions. The present section provides an exploration of some recently developed ID models using machine learning technologies. The IDS emerged in the 1980s for the security of traditional networks against various malicious activities [14]. So far, many security experts and researchers have formulated several IDSs to identify anomalous activities in IoT-enabled smart environments [15]. However, most of the ID models have been developed using machine learning techniques. IDS designed using a machine learning approach provides a promising solution to identify various security issues. Machine learning-based ID approaches are able to recognize malicious patterns in incoming network traffic [16].

Many pieces of research have been conducted so far for threat detection by inspecting the network traffic [17] and classification of events [2]. Authors in [18] presented an ID model to identify unsupervised anomalies and traffic classification using the dataset KDD-Cup99 [19] and real-world network logs for confirmation of the effectiveness of performance. Incremental statistics have been used for feature extraction. Authors in [20] proposed a dataset to incorporate legal and simulated IoT traffic with different types of attacks. Many researchers [20–22] have addressed various existing datasets and evaluated the reliability of the BoT-IoT dataset using statistical machine learning and deep learning approaches for forensics.

IoT networks incorporate the usual network components (laptops, routers, and workstations) with smart IoT devices. Machine-to-machine communication, cloud instances, and popular IoT application providers come up with services in different distributed IoT environments [23]. In [24], the authors analyzed the vulnerable traffic using three machine learning classifiers, namely logistic regression, random forest, and support vector machine (SVM). The authors analyzed the data collected from various IoT devices using different statistical parameters. Data mining, machine learning, and deep learning are the most recent data processing and forecasting approaches which are useful for ID. However, machine learning is the most effective approach due to its better true positive rate (TPR) as compared to other approaches [25]. Rose et al. in [26] explored the prospective of network profiling and monitoring using a dynamic anomaly-based IDS for the investigation of suspected network transactions and potential attacks. Ibrahim et al. [27] compared the performance of the CatBoost classifier with SVM, logistic regression, gradient boosting, AdaBoost [28], random forest, decision tree, K-nearest neighbor (KNN), and many others. Alqahtani et al. [29] proposed a genetic XGBoost-based IDS model using the dimensionality reduction feature selection method to detect botnet attacks on data traffic in IoT, where a publicly available N-BaIoT [30] dataset was used for practical implementation. Tang et al. proposed IDS using light gradient boosting (LGB) and auto-encoder [31]. LGB is one of the most efficient methods of boosting family.

The decision tree is one of the leading approaches of machine learning to make analysis and prediction of intrusions [32]. Decision tree algorithms are supervised machine learning mechanisms that make decisions using bias and variance analysis approaches. Ensemble learning decision tree-based classification model provides more accurate and efficient performance compared to a single decision tree-based classification model. Ensemble methods use the concept of integrating weak learners to attain a strong predictive model to gain better performance. The gradient boosting approach is more promising compared to traditional classification approaches of machine learning [33]. Hyper-parameters are required to be finely tuned to improve the accuracy performance of the model.

Ensemble methods have been implemented in several data mining competitions like KDD-Cup, Netflix Prize, and proposals for the ID framework [34]. Ensemble learning is a learning approach that improves the performance of a machine learning model by incorporating many machine learning models. Most of the winning results of various competitions (like Kaggle) have been in favor of ensemble learning-based approaches. Ensemble methods are commonly used for building ID models due to their feature characterization. Ensembles of different features are combined for a final decision [35]. Ensembles are also used to detect some malicious executables that have never been noticed earlier [36]. It was first implemented by Tianqi Chen but later contributed by many researchers [37]. It relates to a wide collection of tools under Distributed Machine Learning Community (DMLC). XGBoost is one of the best promising ensemble methods that come with competitive outputs [38]. XGBoost allows the tuning of regularization parameters to improve the accuracy, efficiency, and feasibility of the model. Authors in [39] focused on building a strong classification model for IDS. In this article, various decision tree ensemble learning techniques have been analyzed for the prediction of anomalous patterns in the network traffic of IoT systems. In this article, the practical will be carried out using the “TON-IoT” datasets [13].

## 3. Methods and Techniques Used for Proposed Work

Data analysis is an approach to discover useful information by evaluating raw data. It is performed using data analysis tools and a sequence of processes including data cleaning, data transformation, and data modeling. Data analysis helps to make useful predictions and forecasting from big data. There are several techniques to perform data analysis such as traditional techniques, soft computing techniques, and forecasting techniques. The selection of data analysis technique depends on the aim of the investigation and type of data (quantitative or qualitative).

Traditional techniques include regression methods, exponential methods, and least-squares reweighting iterative methods. Traditional techniques are generally used for predictive analysis of small-size datasets for solving simple statistical problems. Some major limitations are associated with the traditional techniques, such as overfitting during processing big data, feature engineering problems, less accuracy, and execution speed. Overfitting and underfitting are the major problems exhibited by statistical models [40]. A statistical model is said to have underfitting when it is not able to capture the underlying logic of the data. It destroys the accuracy of the machine where the model is deployed. It usually happens due to a lack of availability of enough data to build an accurate model. This problem can be overcome by using more data and reducing features in feature selection. Underfitting gives high bias and low variance. On the other hand, a model is said to be overfitted when it is trained with a lot of data. Due to the bulkiness of data in the dataset, the model starts learning with noise and inaccurate data which can lead to the building of unrealistic models. It cannot be avoided completely but can be reduced by reducing the size of the network and appropriate data selection methods [41]. Overfitting has high variance and low bias.

Soft computing techniques include neural networks (ANN), fuzzy logic, knowledge-based expert systems, and genetic algorithms. Soft computing is a new multidisciplinary system that encourages the design of new generation artificial intelligence (AI) to provide solutions to real-world issues. It also motivates the integration of computational tools, techniques, and applications in different combinatorial forms. It is a cost-effective and rapid solution to various complex problems for which solutions do not exist [42]. But it is still a developing and growing technique. Some major limitations are associated with these systems such as high execution time, loss of model interoperability, computational overloading, and limited generalization [43].

Forecasting methods have been focused on for the last few decades for various applications to predict a large number of service points. These can be called as predictive analysis methods. Conventional forecasting and advanced forecasting techniques are commonly used data analysis and forecasting techniques. Conventional methods include simple linear regression, multiple linear regression, and straight-line methods. These methods provide sensible forecasting prediction, but modern forecasting approaches provide much better accuracy as compared to conventional techniques. Moreover, modern techniques have advantages like flexibility, higher efficiency, and interpretability on big datasets. Modern forecasting methods include various machine learning methods such as gradient boosting methods (GBM) and deep learning techniques such as long-short term memory (LSTM). Artificial neural network (ANN) is also a type of advanced forecasting. These techniques provide powerful time-series forecasting and predictive analysis even for big data [44]. This study utilizes machine learning-based modern data analysis methods for constructing and designing the proposed model.

### 3.1. Ensemble Learning

**Figure 1:**
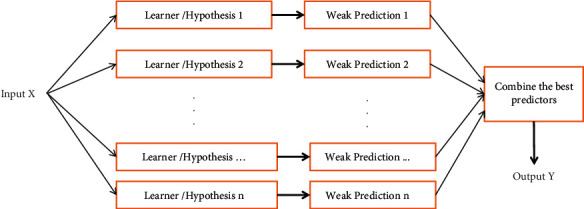
Ensemble-based learning model.

Ensemble approaches have been extensively deployed for application forecasting in various areas due to their simplicity of implementation. Ensemble means to view a group of elements all together instead of using them individually. In the ensemble-based approach, multiple models are created and combined to solve a complex problem [45]. Machine learning algorithms aim to build an unbiased model from a dataset. The constructed model is designated as training or learning, and the model that learns from the data is named as learner or hypothesis.  Figure 1 shows an ensemble-based learning model that assembles a set of classifiers to classify new data points by choosing certain weak predictions to combine them into a strong predictor. Instead of a single classifier, the ensemble methods use a combination of multiple classifiers or predictors which are trained to resolve the same problem and aggregated with each other to obtain better results. Ensemble methods may either use homogeneous base models or different types of base models [43]. Ensemble-based machine learning can optimize the performance of a model by aggregating the prediction results obtained from selected weak models [46]. While aggregating the base models, it is required that a base model with high variance and low bias must be aggregated using a variance reducing scheme, and a base model with high bias and low variance must be aggregated using a bias reducing scheme [47].

Most of the traditional learning approaches which generate a single hypothesis face many computational, statistical, and representational problems which could be conquered by ensemble learning to some extent [48]. The resulting prediction of the ensemble is obtained through majority voting [49]. These techniques generally depend on randomization approaches, which are able to generate manifold solutions to imminent problems. Ensemble assists to upgrade the generalization and robustness of the model. Decision tree-based traditional learning methods face high variance and bias problems. Actually, the bias-variance trade-off is the basic property of a predictive model. Bias occurs due to wrong belief in the algorithm and high bias indicates the underfitting of the model. On the other hand, the variance occurs due to the sensitivity of the algorithm and indicates that the model is too complex, and it leads to overfitting. Hence, there must be a proper balance between bias and variance in an ideal model. An individual decision tree generates a single hypothesis, and an ensemble of decision trees can produce much better results by reducing bias and variance [48]. Bagging and boosting are most widely used ensemble approaches [50] that will be discussed further in this section.

#### 3.1.1. Bagging

Bagging is also recognized as bootstrap aggregation, and it uses sequential as well as parallel methods to generate samples. Bagging generally uses analogous weak learners and trains them concurrently, followed by combining them using certain averaging methods. In bagging, multiple base learners are hypothesized on a randomly selected set of training instances with replacement, and a base learner is trained on each set [51]. Bagging follows “voting” and “regression averaging” methods for solving the classification problems. Random forest (RF) is an example of bagging that is widely used in design of an IDS [52]. The structure of bagging algorithm is very much similar to the structure of general ensemble learning and has been shown in Figure 1.

*(1)*. *Random Forest (RF)*. Random forest is one of the most successful and well-known ML algorithms that is known for high accuracy and independent fast learning over datasets of distinct nature. In [49], Breiman proposed a value for this parameter which is “[log_2_ (#f) + 1]”, where “f” is the set of features. Random forest algorithm is an ensemble approach that makes predictions on the basis of results obtained from a group of decision trees. It performs the resampling of trees using the bootstrap (bagging) mechanism [53]. The trees in a forest are trained using a bootstrap subset created for training. Each node in RF is split using the best predictor that is chosen randomly at the node level. The additive random layer makes it stronger against overfitting. A small de-correlating twist is made to improve the bagged trees. Many decision trees could be built by bagging on bootstrap sets of training data. A random sample of n predictors is selected as a splitting candidate out of a complete set of predictors. The number of trees in the forest becomes larger, so the generalization error of random forest meets a limit. The major advantage of random forest is that parameters are rarely required to be tuned and remarkable results could be obtained through default parameter settings [54]. Here, the parameters that need to be tuned are related to controlling the depth of the decision tree. The growth of the decision tree could be bounded by tuning the “maximum depth” and “number of instances per node.”

Random Forest (Bagging) Algorithm

  Step1: Select P random data points (random samples) from the training set of the given dataset.
  Step2: Build the decision tree using selected data points for every point.
  Step3: Specify m, the number of decision trees to be built.
  Step4: Repeat steps 1 and 2.
  Step5: Find the predictive value of each decision tree and the data points to the winner of majority voting.

Advantages of Random Forest

- RF has the potential to solve both classification and regression problems.
- RF can improve the accuracy of the model.
- RF is less liable to the problem of overfitting.

Disadvantages of Random Forest

- Computations may become more complex due to the high number of trees.
- The algorithm may make modifications by minor data transformation.

#### 3.1.2. Boosting

Boosting is a technique used for improving the accuracy of a learning model. Boosting adopts the sequential ensemble method [55]. Using the boosting technique, the ensemble learner can boost the weak learner and convert into strong learner [50]. A strong learner is an optimized learner that approaches nearly perfect (moderate) performance. The idea behind boosting is to reduce classification errors and improve the results over many other classification algorithms. A set of learners are trained sequentially and merged for prediction. Each base model depends on the previous base model.

**Figure 2:**
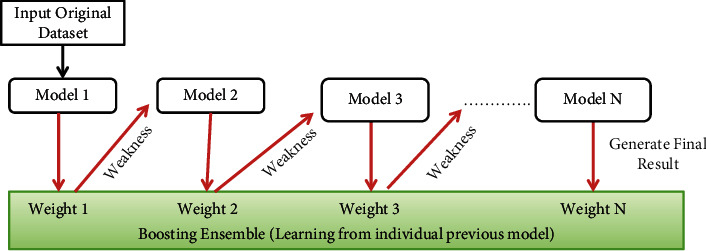
Boosting ensemble-based learning model

Machine learning models designed using boosting algorithms emphasize the premium quality prediction done by a single model. Models designed using boosting methods produce superior results [56]. A specific weak model can be improved using the boosting mechanism. The boosting algorithm attempts to enhance the prediction potential by training a series of weak models. Each next individual model is trained with the input data and the weakness of its previous model and attempts to recover the deficiency of its predecessor. A model developed using the boosting technique is recognized as a generic model instead of the specific model. The idea behind boosting is to design an efficient algorithm to convert relatively weak hypotheses into very strong hypotheses. These strong learners of boosting algorithms are also faster than the learners of bagging (random forest). Actually, boosting algorithm boosts the performance of classification as well as regression [57].  Figure 2 shows the boosting ensemble-based learning model, where each individual model learns on the weakness of its previous model.

Boosting Algorithm

  Step 1: Train the first base model (say model 1) with input Dataset D and the learning algorithm.
  Step 2: Calculate the result in the form of weight.
  Step 3: Train the next base model with the weak predicted result of its previous model and repeat step 2.
  Step 4: Repeat step 3 until model N.
  Step 5: Obtain weight N as the final prediction and generate the final result.

*(2) Gradient Boosting (GB)*: AdaBoost was later generalized as GB. In AdaBoost, the weak learner refers to the decision tree with a single split called decision stumps. GB is one of the most powerful decision tree algorithms of machine learning. Prediction models built using GB give outstanding accuracy and speed in the case of large and complex datasets (a large number of features and/or samples) [57]. Bias and variance are two significant errors that are solved by machine learning-based models. GB ensemble models reduce bias errors very efficiently. Gradient boosting algorithm integrates a number of weak classifiers to make a strong classifier F(x) [29]. In the GB approach, the classification depends on the residuals of the previous iterations. Consider a training dataset D; where D = {X_i_y_i_}_1 ..,..N_, and the objective of GB is to get an approximation. Gradient boosting constructs an additive approximation of the weighted sum of functions (*F*^*∗*^(*X*)) that has been presented as follows:

(1) $$\begin{array}{c} F^{*}\left(X\right)=F_{k-1}\left(X\right)+\rho_{k}h_{k}\left(X\right), \end{array}$$

where *ρ*_*k*_ is the weight of the *k*^th^ function *h*_*k*_ (*X*). The approximation is built iteratively; for which constant approximation, *F*_0_(*X*) is obtained initially for *F*^*∗*^(*X*). The functions are the models of an ensemble technique. If the iterative process is not regularized properly, the built model can face the overfitting problem. There are many regularization parameters that can be considered to control the GB additive process. Gradient boosting can be regularized naturally using the shrinkage process to reduce every step of the gradient descent *F*^*∗*^(*X*). The following equation introduces the shrinkage into GB using regularization parameters *ν* and k:

(2) $$\begin{array}{c} F_{k}\left(X\right)=F_{k-1}\left(X\right)+\nu \rho_{k}h_{k}\left(X\right),0<\nu \leq 1, \end{array}$$

where parameter *ν* is the “learning rate” and *k* represents the number of components. These regularization parameters can control the degree of fit that affects the result optimality. Increasing or decreasing the value of learning rate influences the outcome. The effect of every feature is calculated sequentially in order to obtain target accuracy [45].

Loss function L(*φ*) is used to calculate the residuals. The loss function is optimized using gradient descent. Final result Ø(*X*) is calculated by adding the results of the T sequential classifiers. *f*_*k*_ is the decision tree and M is the total number of iterations [58]. The following equation presents the mathematical calculation of final result of GB:

(3) $$\begin{array}{c} Y=Ø\left(X\right)=\sum_{k=1}^{M}f_{k}\left(X\right), \end{array}$$

where *f*_*k*_ ∈ F. Gradient boosting method requires the following three main components: loss function optimization, prediction using weak learner, and an additive model to add a weak learner for minimization of a loss function. This algorithm incorporates many weak learners into a strong learner in a repetitive manner [59].

Advantages of Gradient Boosting

- Gradient boosting is a greedy algorithm that can quickly overfit the training dataset.
- Performance of the algorithm can be improved by reducing the overfitting.
- Using the regularization approach, various parts of the algorithm can be panelized.

Disadvantages of Gradient Boosting

- High running rate, power consumption, memory usage, and training time.
- Interpretability problem.

*(3)*. *Extreme Gradient Boosting (XGB)*: Extreme gradient boosting or XGBoost is a decision tree-based improved GB algorithm that can improve the accuracy, efficiency, and feasibility of ensemble-based IDS [60]. It can smoothly deal with bias-variance trade-offs. It is recently being dominated by prediction problems including unstructured data (text, images, voice). It can be used to solve a wide range of problems including classification, regression, user-defined prediction, and ranking. It performs parallel computation at the node level that makes it faster and more powerful than GB [61]. Many researchers have proved it as one of the fastest and memory-efficient machine learning algorithms. XGBoost as an anomaly detection system gives superior performance. The following equation shows the mathematical explanation of XGBoost:

(4) $$\begin{array}{c} F\left(X,w\right)=\sum_{k=0}^{K}\alpha_{k}h_{k}\left(X,w_{k}\right)\\ =\sum_{k=0}^{K}f_{k}\left(X,w_{k}\right). \end{array}$$

The main aim of XGB is residual fitting. Residual is the difference between real and predicted values. Here, *X* is the input data, F(X, w) is the model to be obtained, *h*_*k*_ is used for a single tree, *w* is the tree's parameter, and *α*_*n*_ is weight of k number of trees. We can obtain the optimal model by minimizing the loss function *F*^*∗*^ [38]. XGBoost also follows the randomization technique to improve the performance of training speed and to reduce the overfitting. The randomization technique in XGBoost contains the following four major hyper-parameters: column subsampling of a tree and its node levels; random subsamples for training independent trees; learning rate; and n estimators. XGBoost is a convenient algorithm to construct a robust classification model. Due to various features, it can efficiently deal with many issues related to data classification and high-level preprocessing [39]. It is able to convert a weak (hypothesis) learner into a strong (hypothesis) learner using the optimization process. By adding every new tree enables the classification model to develop fewer false alarms, accurate data classification, and easy data labeling [62].

Advantages of XGBoost

- XGB is comparatively faster than other existing boosting algorithms.
- It contains linear as well as tree learning algorithms.
- XGB library is mainly used to design faster and highly efficient decision tree models.
- It reduces the computing time and optimally utilizes the memory.
- It performs parallel processing. It can use all cores of the device it is executing on.
- Regularization is a significant feature that enables it to reduce overfitting problems.
- Portability and flexibility are the important features of this algorithm.
- XGB is able to convert a weak learner into a strong learner using its optimization process.
- It can efficiently detect and handle missing and null values.
- In XGB, tree pruning continues to the maximum depth.
- XGB can utilize the resources efficiently.

Disadvantages of XGBoost

- Although it has a simple solution, it is still not convenient to optimize memory usage.
- XGB takes high execution time.

*(4)*. *Light Gradient Boosting Method (LGBM)*

LGBM is a novel boosting model which was proposed by Microsoft in 2017. The outcomes of various machine learning techniques and the results of ensemble-based techniques are tested with various parameters such as accuracy and speed. LGB is a distributed, quick, and high-performance gradient-based uplifting algorithm that is derived from popular machine learning algorithms [63]. Samples with small gradients are well trained (sometimes generate a small error in training) and those with large gradients are undertrained. This algorithm expands leaf-wise instead of node-wise and the maximum delta value is chosen for leaf-wise augmentation. It can be used for solving many machine learning problems like regression, classification, and prediction [58, 64]. The process of bucketing continuous features into discrete bins increases the training speed. This factor also improves the efficiency. It follows the leaf-wise split method instead of level-wise, which results in much more complex trees. This factor plays a role in attaining higher accuracy. However, it can lead to an overfitting problem which can be prevented by tuning the parameter “max_depth”. LGB is composed of decision trees which are constructed using the following procedure.

The method to calculate the gain of variation occurs under weak and strong gradients. The training samples are organized in decreasing order as per the absolute value of their big and small gradients (*g*_*i*_). The first s% samples with bigger gradients are preserved to construct the subset of samples S. The remaining set S^C^ is created by the (1 − *s*) % of samples with smaller gradients [31]. The subset R with size *r*^*∗*^| S^C^| is constructed randomly. Finally, the samples are divided in accordance with the evaluated *V*_*j*_^*∗*^(d) (variance gain) on S ∪ R subset. Equation (5) presents the mathematical formula for variance gain. Let the feature set be *x*_*i*_ , where *x*_1_, *x*_2_, *x*_3_,……*x*_*n*_

(5) $$\begin{array}{c} V_{j}*\left(d\right)=\frac{1}{n}\left(\frac{{\left(\sum x_{i}\in S_{a}g_{i}+\left(1-s/r\right)\sum x_{i}\in R_{a}g_{i}\right)}^{2}}{n_{a}^{j}\left(d\right)}+\frac{\left(\sum x_{i}\in S_{b}g_{i}+\left(1-s/r\right)\sum x_{i}\in R_{b}g_{i}\right){}^{2}}{n_{b}^{j}\left(d\right)}\right), \end{array}$$

where *S*_*a*_={*x*_*i*_ ∈ *S* : *x*_*ij*_ ≤ *d*}, *S*_*b*_={*x*_*i*_ ∈ *S* : *x*_*ij*_ > *d*}, *R*_*a*_={*x*_*i*_ ∈ *R* : *x*_*ij*_ ≤ *d*}, and *R*_*b*_={*x*_*i*_ ∈ *R* : *x*_*ij*_ > *d*}; *d* is the point of partitioning the dataset to calculate the best gain invariance; and 1 − *s*/*r* is used for normalizing the gradient sum over R. Each feature in *x*_*i*_ is utilized to calculate the split of training data covering all trees. Important hyper-parameters of LGB are “learning_rate”, “max_depth”, and “n_estimators” which could be tuned to obtain the best performance of the model. LGB uses exclusive feature bundling and gradient-based one-side sampling (GOSS) for faster processing [65].

Advantages of LGBM

- LGB is the fastest among all decision tree-based algorithms.
- It serves the fastest training speed and reduces computational complexity.
- In LGB, continuous values are replaced by discrete bins resulting in less memory consumption.
- It consumes low communication cost.
- It performs with good accuracy.
- Less time consumption for data preprocessing and decision-making is the most significant feature of LGB.

Disadvantages of LGBM

- Sometimes it compromises in accuracy.

*(5) CatBoost (CB)*. CatBoost is a novel open-sourced GB library that strongly deals with categorical features even during the time of preprocessing [27]. It is used as a new framework for leaf value calculation while choosing the tree schema, which helps to minimize overfitting. It gives good performance in terms of accuracy. Decision trees are suitable for datasets containing numerical features. However, datasets that contain categorical features cannot be predicted by a decision tree. Such features contain discrete sets of values such as name and ID, which are not comparable with each other. CatBoost is feasible for such features that convert categorical data to numerical data before training while preprocessing [66]. The main hyper-parameters of CatBoost are given in Table 1.

Advantages of CatBoost

- High training and test accuracy.

Disadvantages of CatBoost

- It compromises in speed.

There are certain key differences between bagging and boosting ensemble algorithms which have been identified by exploration of the existing literature and research work done earlier in this section (see Table 2). The next section identifies the differences between bagging and boosting-based algorithms on the basis of practical analysis.

## 4. Dataset Selection and Practical Implementation

Several methods of machine learning have been used so far for anomaly detection considering binary classification with different experimental setups [66–71]. In many cases, they have achieved outperforming results. In this section, the study explores and analyzes the performance of bagging and boosting algorithms to identify the best algorithm for ID model considering the multiclass classification of the “TON-IoT dataset” which consists of further datasets related to individual IoT home scenarios. Each dataset has a specific number of features and number of instances (see Table 3). Through experiments, the study predicts the nature of the individual record in a dataset means whether it is normal or anomalous. This study utilizes ensemble-based bagging and boosting techniques that are trained on IoT datasets of certain home scenarios. Each dataset has 7 or 8 multiclass labels which will be further classified using ensemble-based classification techniques [72].

### 4.1. Procedure for Data Computing and Analysis

The entire computing and data analysis procedure will be implemented using python programming in the jupyter notebook: An interactive computing environment.

- Identify and adopt a dataset suitable for ID problems that contain the records of network traffic of IoT environment.
- Download the “CSV” file containing the “TON-IoT” dataset that will be utilized as an array.
- Load and prepare data to train and evaluate a model. Data will be prepared using certain preprocessing techniques, namely, data cleaning, data transformation [73], scaling, and feature engineering [74].
- Split the dataset array (features or attributes) into X (input) and Y (output). Specify the attribute indices in the format of the NumPy array. After analyzing the significance of features, select the most promising features to compute the output [75]. The feature selection method removes the noisy data and improves the performance of the classifiers [76].
- Split the X and Y data into training and test data. The training data will be used to prepare and train a model and test data will be used to make predictions. Specify the size of test data. Calculate Y_prediction using scikit learn method “model.predict()”.
- Train the model using different ensemble-based classification algorithms of machine learning.

## 5. Experimental Results and Discussion

In this section, the results have been figured out by implementing the ensemble-based machine learning approach on training and test data of TON-IoT datasets based on IoT sensors. Generally, the test_size is taken as 20% to 35% of the total data and the rest of the data is used to train the model. Each individual ensemble algorithm has its classifier method that is utilized for designing the model. Table 1 presents the details of various classifiers considered in this article.

Performance of bagging and boosting algorithms could be optimized by tuning their respective hyper-parameters. The values are assigned to the hyper-parameters of each classifier and tested until they reach to their best performance. Hyper-parameters of XGB could be more finely tuned as compared to other classifiers.

### 5.1. Selection of Ensemble Algorithms

The algorithm for model designing will be selected on the basis of analysis and results obtained using different parameters. Different bagging and boosting algorithms will be examined and validated on couple of important metrics such as accuracy score, speed, precision, recall, F1-score, and mean accuracy.

Table 4 shows the computed results of the train and test accuracy of ensemble bagging (random forest) and ensemble GB (XGB, LGB, and CB) classifiers. The accuracy performance depends on train_test_split parameters such as “test_size” and “random_state”. The performance has been evaluated on data extracted from different IoT devices (fridge, garage door, GPS tracker, motion light, thermostat, and weather monitoring system) in a smart home environment. In different cases, the accuracy scores of the examined algorithms slightly vary from each other. Accuracy is a prime metric to compare ML-based models, and a good model must attain high accuracy. However, it is a necessary condition, not sufficient. The algorithm must be evaluated on a number of parameters to validate and prove its efficiency.

### 5.2. Evaluation Metrics

Along with the accuracy, some other metrics (true positive rate (TPR), false positive rate (FPR), precision, recall, F1-score) also have been utilized to evaluate the performance of the algorithms for the proposed ID model. Accuracy is the percentage of correctly classified anomalous and the normal index. TPR and TNR are the percentages of correctly classified total relevant detection rates. The following equation presents the formula for mathematical calculation of accuracy:

(6) $$\begin{array}{c} \text{Accuracy}=\frac{\text{TP}+\text{TN}}{\text{TP}+\text{FN}+\text{TN}+\text{FP}}. \end{array}$$

- True positive (TP) refers to the number of the actual threats which have been classified as threats. It means the predicted and actual classes are the same and true.
- True negative (TN) refers to the normal events which have been classified as normal. It means the predicted and actual classes are the same but false.
- False positive (FP) refers to the number of normal events misclassified as intrusions. It means the predicted and actual classes are not the same and they are true and false, respectively.
- False negative (FN) refers to the number of intrusions misclassified as normal. It means predicted and actual classes are not the same and they are false and true, respectively [77]. It shows that some threats in the IoT environment have not been predicted. This is known as “unbalanced classification” [78].

Just measuring the accuracy with good results is not sufficient to prove it a most efficient algorithm; still, there are chances that the model will predict false negative values.

(7) $$\begin{array}{c} \text{TPR or Sensitivity or Recall}=\frac{\text{TP}}{\text{TP}+\text{FN}}, \end{array}$$

(8) $$\begin{array}{c} \text{FPR or }\left(1-\text{Specificity}\right)=\frac{\text{FP}}{\text{TN}+\text{FP}}. \end{array}$$

TPR or recall or sensitivity determines that how many relevant instances have been selected. TPR and TNR determine the percentage of total relevant attack vectors and normal events, respectively, which have been correctly classified by the classifier. TPR and FPR are the detection rates, where TPR is the actual positive rate and the FPR is the actual negative rate.

The precision or specificity determines the percentage of relevant outcomes that means how many instances are relevant out of the total selected instances. Equations (9) and (10) refer to the mathematical formula of Precision and F1-score, respectively. F1-score refers to the harmonic mean of precision and recall. It might be due to disproportionate class distribution in the training dataset.

(9) $$\begin{array}{c} \text{Precision}=\frac{\text{TP}}{\text{TP}+\text{FP}}, \end{array}$$

(10) $$\begin{array}{c} \text{F}1\text{\_score}=2*\frac{\text{Precision}*\text{Recall}}{\text{Precision}*\text{Recall}}. \end{array}$$

Here, the precision, recall, and F1-score of different algorithms have been computed followed by the analysis of the classification reports of different decision tree classifiers for the considered datasets.  Table 5 shows that in most cases, boosting-based algorithms result in the highest classification scores. But no specific boosting algorithm produces the highest classification score for all the considered datasets and this analysis could not prove the highest efficiency of any algorithm. Hence, this evaluation could not do enough work to identify the most efficient algorithm for classification and prediction model for ID.

Now this study examines the mean accuracy of considered datasets using earlier discussed algorithms and some more GB (HistGradient boosting) algorithms to validate their efficiency (see Table 6). The mean accuracy score evaluation has been performed using “kFoldCrossValidation” method of “sklearn.model_selection” module of sklearn library with random sets of train and test data. It is obtained by calculating the average of k recorded accuracy. It also serves as a performance metric of the model that validates the performance more strongly. This is a method to train and test the model on a different set of samples instead of repeating the same data sample. By selecting the value of k, one can estimate the skill of the model on random partitions of the original data. Here, the “RepeatedStratifiedKfold” method of cross-validation has been utilized with parameters “n_splits”, n_repeats”, and “random_state” to obtain the prediction accuracy. Table 6 shows that using the k-fold cv, the accuracy of XGBoost and CatBoost was found better than other comparative algorithms. The parameters have been tuned to obtain the highest accuracy. Here, the code has been executed with different values assigned to the hyper-parameters. The values of “n_splits = 5” and “n_repeats = 3” have been assigned and the results produced by CatBoost algorithm are highest in accuracy. 

Table 7 shows the runtime consumed by various ensemble-based classification algorithms examined in this study. Runtime is the execution time that represents the total time consumed by an algorithm from start to stop. If the data size is big, then it will consume time in seconds. It is very much important to select an ideal algorithm that can execute the problem in minimum time duration. For example, a model has been designed for the classification of records for prediction of crime, and if it is taking too much execution time, then it might lead to a delay in crime investigation. It may also cause destruction and theft of pieces of evidence during the delay time. Hence, runtime is also an important parameter to select an algorithm. Here, the LGB classifier has taken the minimum time (in seconds) that is many times less compared to some other algorithms. Therefore, an ID model designed using LGB can give the best possible performance in terms of speed.

**Figure 3:**
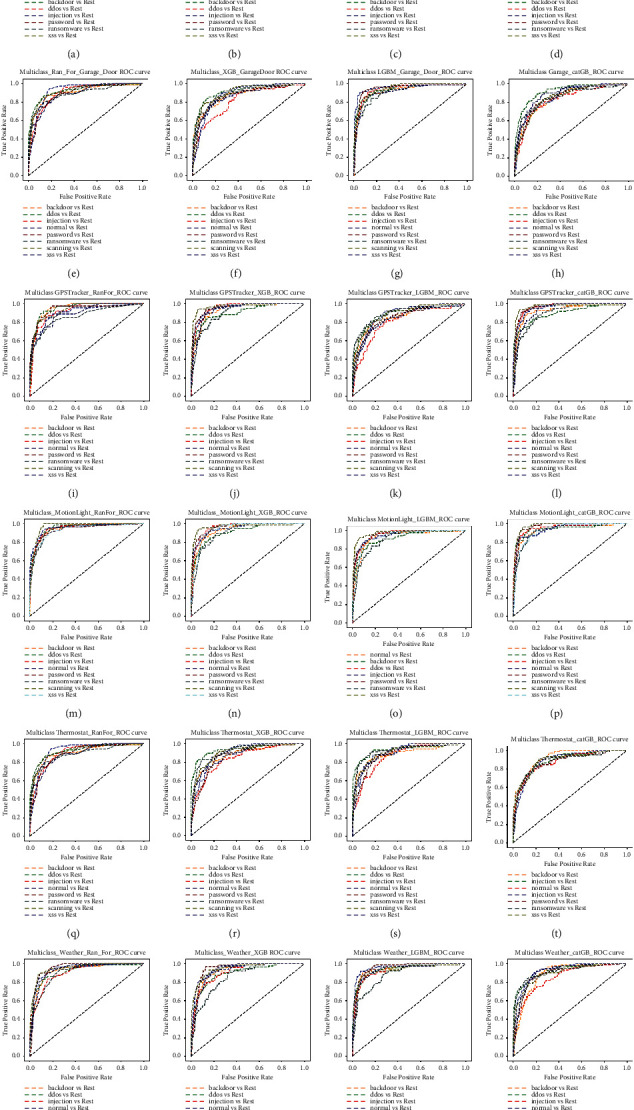
ROC_AUC curves of classification models of datasets: (a) Fridge using random forest, (b) Fridge using XGBoost, (c) Fridge using LGBM, (d) Fridge using CatBoost, (e) Garage_Door using random forest, (f) Garage_Door using XGBoost, (g) Garage_Door using LGBoost, (h) Garage_Door using CatBoost, (i) GPS_Tracker using random forest, (j) GPS_Tracker using XGBoost, (k) GPS_Tracker using LGBoost, (l) GPS_Tracker using CatBoost, (m) Motion_Light using random forest, (n) Motion_Light using XGBoost, (o) Motion_Light using LGBoost, (p) Motion_Light using CatBoost, (q) Thermostat using random forest, (r) Thermostat using XGBoost, (s) Thermostat using LGBoost, (t) Thermostat using CatBoost, (u) Weather using random forest, (v) Weather using XGBoost, (w) Weather using LGBoost, and (x) Weather using CatBoost.

Figures 3(a)to 3(x) present the ROC-AUC curves for the “TON_IoT” dataset in different home scenarios. The curves measure the correctness of the rank order of classification [34]. The results have been represented using the receiver operating characteristic (ROC) curve that shows the graph of the performance of a classification framework at each classification threshold. These are important aspects of machine learning for graphical representation of true positive (actual positive) rate and false positive (actual negative) rate. ROC curves are generated by plotting the connection (trade-off) between TPR (recall) and FPR on distinct threshold locations. ROC curves are used to test and compare the adequacy of a model. Figures 3(a)to 3(d) present the TPR and FPR for IoT_Fridge; Figures 3(e)to 3(h) present the TPR and FPR for IoT_Garage_Door; Figures 3(i)to 3(l) present the TPR and FPR for IoT_GPS_Tracker; Figures 3(m)to 3(p) present the TPR and FPR for IoT_Motion_Light; Figures 3(q)to 3(t) present the TPR and FPR for IoT_Thermostat; and Figures 3(u)to 3(x) present the TPR and FPR for IoT_Weather using random forest, XGB, LGBM, and CatBoost classifiers.

Table 8 presents the ROC_AUC score that is computed using the ROC_AUC_Score () method whose parameters are “y”, “predict_proba(X)”, and “multi_class = ovr”. The predict_proba(X) calculates the probability of the types of output classes on the basis of input samples. The performance of the classification results generally depends on some hyper-parameters of “make_classification” function, namely, “Number of samples”, “selected features”, and “random states”. Here “ovr” stands for One-Vs-Rest that is used for multiclass classification. It divides the multiclass dataset into numerous binary classification problems. AUC presents the relation between TPR and FPR. The highlighted values in Table 8 show that the boosting classifier, especially LGB, produces highest ROC_AUC score in most of the cases.

The ROC_AUC curves in Figures 3(a)to 3(x) show that the random forest and the light gradient boost (LGB) algorithms have the highest TPR and FPR. LGB takes the lowest runtime and high TPR and FPR. Sometimes LGB compromises in the accuracy but not too much. The accuracy results produced by LGB are not very much less than others. Runtime must also be considered for selecting an efficient predictive algorithm in designing a model for an IDS.

## 6. Conclusion

In this article, the ensemble-based machine learning algorithms handled very complex and big data of Internet of Things with good potentiality. The ensemble bagging and boosting classification approaches have been analyzed to retain a good multiclass classification model that can predict the types of normal and anomalous classes in IoT network traffic. This article also has validated the algorithms for designing a model for intrusion detection by training on several sets of train data and evaluated on separate test data by tuning the values of hyper-parameters. For avoiding the repetitions, each time the training and test sets have been shuffled using “RepeatedStratifiedKfold” methods of cross-validation. In this article, the comparison of ensemble bagging and boosting algorithms has been carried out in terms of accuracy score, mean accuracy score, speed, precision, recall, F1-score, and auc_score. The light gradient boosting (LGB) algorithm is the most efficient algorithm in terms of speed and ROC_AUC score. Sometimes LGB compromises in terms of accuracy. Still, the accuracy score is very good, and it is not very much less than other classification algorithms. Random forest is also one of the most accurate algorithms but it takes high execution time and computational power and it is too much complex. Speed is an important metric for an intrusion detection model to obtain quick outcomes and LGB was found to be the fastest algorithm with lowest runtime. Hence, light gradient boost is the best algorithm to be selected for designing an efficient intrusion detection system. In future, the proposed work will be helpful to design a very fast, accurate, and lightweight intrusion detection model for IoT-based smart environment. The proposed work will be helpful to perform multiclass classification of datasets containing large number of complex data records. There is a wide scope of the proposed work not particularly for intrusion detection in IoT but also for classification and prediction of various applications of different environments.

## Figures and Tables

**Table 1:** Details of classifiers.

Algorithm	Classifier	Tuned hyper parameters
Random forest	RandomForestClassifier(….)	Random_state, n_jobs, max_depth; n_estimators, criterion = “entropy”
Extreme gradient boosting	XGBClassifier(….)	random_state, n_estimators, max_depth, learning_rate, eval_metric = “mlogloss”
Light gradient boosting	LGBMClassifier(….)	Random_state, n_estimators, num_leaves, max_depth
CatBoost	CatBoostClassifier(…..)	learning_rate, iterations, max_depth, loss_function = “Multiclass”

**Table 2:** Summarized differences between Bagging and Boosting.

Bagging	Boosting
Weak models often learn independently in parallel	Weak models often learn sequentially in an adaptive way
Bagging focuses on obtaining an ensemble model with less variance	Boosting focus on producing a strong model with less bias but variance can also be reduced
Different weak learners can be fitted independently and train concurrently	Different weak learners cannot be fitted independently but models are fitted iteratively and training of each model depends on the model fitted previously
The idea behind boosting is to construct a set predicting model by aggregating the results of base models.	The idea behind boosting is to construct a set of models which are aggregated to get a strong learner.

**Table 3:** Specification of TON_IoT datasets [13].

Datasets	Features	No. ofinstances	Input features	Output feature = “type” (classes)
TON_IoT (IoT_Fridge)	6	587077	“Date”, “time”, “fridge_temperature”, “temp_condition”, “label”	“Normal”, “backdoor”, “ddos”, “injection”, “password”, “ransomware”, “xss”
TON_IoT (IoT_Garage_Door)	6	591447	“Date”, “time”, “door_state”, “sphone_signal”, “label”	“Normal”, “backdoor”, “ddos”, “password”, “injection”, “scanning”, “ransomware”, “xss”
TON_IoT (IoT_GPS_Tracker)	6	595687	“Date”, “time”, “latitude”, “longitude”, “label”	“Normal”, “backdoor”, “ddos”, “injection”, “password”, “ransomware”, “scanning”, “xss”
TON_IoT (IoT_Motion_Light)	6	452263	“Date”, “time”, “motion_status”, “light_status”, “label”	“Backdoor”, “ddos”, “injection”, “normal”, “password”, “ransomware”, “scanning”, “xss”
TON_IoT (IoT_Thermostat)	6	442229	“Date”, “time”, “current_temperature”, “thermostat_status”, “label”	“Backdoor”, “injection”, “normal”, “password”, “ransomware”, “scanning”, “xss”
TON_IoT (IoT_Weather)	7	650243	“Date”, “time”, “temperature”, “pressure”, “humidity”, “label”	“Normal”, “backdoor”, “ddos”, “injection”, “password”, “ransomware”, “scanning”, “xss”

**Table 4:** Accuracy score.

Dataset name	Random forest		XGBoost		LGBM		CatBoost	
	Train	Test	Train	Test	Train	Test	Train	Test
TON_IoT (IoT_Fridge)	91.36	91.36	91.37	91.36	91.36	91.35	91.38	91.37
TON_IoT (IoT_Garage_Door)	93.16	93.14	93.16	93.14	93.16	93.14	93.16	93.14
TON_IoT (IoT_GPS_Tracker)	92.94	92.91	97.26	97.28	94.89	94.74	94.18	94.07
TON_IoT (IoT_Motion_Light)	92.08	92.16	92.09	92.11	92.09	92.11	92.09	92.11
TON_IoT (IoT_Thermostat)	95.31	95.32	95.33	95.32	95.31	95.32	95.32	95.32
TON_IoT (IoT_Weather)	96.69	96.69	96.49	96.26	96.91	96.80	95.84	95.84

**Table 5:** Classification report.

Dataset	Models	Precision	Recall	F1-score
TON_IoT (IoT_Fridge)	Random forest	0.89	0.91	0.89
	XGB	0.89	0.91	0.89
	LGBM	0.89	0.91	0.89
	CB	0.90	0.91	0.89

TON_IoT (IoT_Garage_Door)	Random forest	0.90	0.93	0.91
	XGB	0.90	0.93	0.91
	LGBM	0.90	0.93	0.91
	CB	0.90	0.93	0.91

TON_IoT (IoT_GPS_Tracker)	Random forest	0.92	0.93	0.92
	XGB	0.97	0.97	0.97
	LGBM	0.95	0.95	0.95
	CB	0.94	0.94	0.94

TON_IoT (IoT_Motion_Light)	Random forest	0.89	0.92	0.90
	XGB	0.89	0.92	0.90
	LGBM	0.89	0.92	0.90
	CB	0.89	0.92	0.90

TON_IoT (IoT_Thermostat)	Random forest	0.92	0.95	0.94
	XGB	0.93	0.95	0.94
	LGBM	0.92	0.95	0.94
	CB	0.92	0.95	0.94

TON_IoT (IoT_Weather)	Random forest	0.97	0.97	0.96
	XGB	0.96	0.96	0.96
	LGBM	0.97	0.97	0.97
	CB	0.96	0.96	0.96

**Table 6:** Mean accuracy (cross validation).

Dataset name	Mean accuracy (Cross_Validation)					
	Random forest	Gradient boosting	HistGradient boosting	XGB classifier	LGBM classifier	CB classifier
TON_IoT (IoT_Fridge)	94.3	92.0	93.8	94.3	93.9	94.5
TON_IoT (IoT_Garage_Door)	93.9	91.1	93.6	93.9	93.6	94.4
TON_IoT (IoT_GPS_Tracker)	94.3	92.0	93.9	94.0	93.9	94.5
TON_IoT (IoT_Motion_Light)	95.2	92.6	94.8	95.2	94.8	95.4
TON_IoT (IoT_Thermostat)	93.7	91.8	93.3	93.3	93.4	93.8
TON_IoT (IoT_Weather)	95.4	93.1	95.4	95.4	95.4	95.7

**Table 7:** Runtime (in seconds) performance.

Dataset name	Runtime (in seconds)					
	Random forest	Gradient boosting	HistGradient boosting	XGB classifier	LGB classifier	CB classifier
TON_IoT (IoT_Fridge)	39.935	41.937	8.119	43.124	6.495	113.525
TON_IoT (IoT_Garage_Door)	43.233	54.916	9.368	50.577	5.997	124.883
TON_IoT (IoT_GPS_Tracker)	40.104	46.075	8.329	43.663	5.966	114.045
TON_IoT (IoT_Motion_Light)	45.709	52.981	12.657	51.432	5.019	132.642
TON_IoT (IoT_Thermostat)	49.82	36.902	6.753	36.856	5.447	95.517
TON_IoT (IoT_Weather)	51.05	61.381	8.928	53.552	6.729	134.768

**Table 8:** ROC_AUC_Score.

Dataset name	ROC_AUC score			
	Random forest	XGB classifier	LGB classifier	CB classifier
TON_IoT (IoT_Fridge)	98.57	98.70	98.65	98.74
TON_IoT (IoT_Garage_Door)	98.57	98.74	98.95	98.99
TON_IoT (IoT_GPS_Tracker)	98.23	98.26	98.69	98.39
TON_IoT (IoT_Motion_Light)	98.80	98.91	98.99	98.86
TON_IoT (IoT_Thermostat)	98.57	98.63	99.33	98.75
TON_IoT (IoT_Weather)	98.78	98.73	99.23	99.03

## Data Availability

The data are available on request from the corresponding author.
